# Development and validation of algorithms to build an electronic health record based cohort of patients with systemic sclerosis

**DOI:** 10.1371/journal.pone.0283775

**Published:** 2023-04-13

**Authors:** Ann-Marcia C. Tukpah, Jonathan A. Rose, Diane L. Seger, Paul F. Dellaripa, Gary M. Hunninghake, David W. Bates

**Affiliations:** 1 Division of Pulmonary and Critical Care Medicine, Department of Medicine, Brigham and Women’s Hospital, Boston, Massachusetts, United States of America; 2 Division of General Internal Medicine and Primary Care, Department of Medicine, Brigham and Women’s Hospital, Boston, Massachusetts, United States of America; 3 Division of Rheumatology, Inflammation and Immunity, Department of Medicine, Brigham and Women’s Hospital, Boston, Massachusetts, United States of America; University of Manchester School of Biological Science: The University of Manchester Faculty of Biology Medicine and Health, UNITED KINGDOM

## Abstract

**Objectives:**

To evaluate methods of identifying patients with systemic sclerosis (SSc) using International Classification of Diseases, Tenth Revision (ICD-10) codes (M34*), electronic health record (EHR) databases and organ involvement keywords, that result in a validated cohort comprised of true cases with high disease burden.

**Methods:**

We retrospectively studied patients in a healthcare system likely to have SSc. Using structured EHR data from January 2016 to June 2021, we identified 955 adult patients with M34* documented 2 or more times during the study period. A random subset of 100 patients was selected to validate the ICD-10 code for its positive predictive value (PPV). The dataset was then divided into a training and validation sets for unstructured text processing (UTP) search algorithms, two of which were created using keywords for Raynaud’s syndrome, and esophageal involvement/symptoms.

**Results:**

Among 955 patients, the average age was 60. Most patients (84%) were female; 75% of patients were White, and 5.2% were Black. There were approximately 175 patients per year with the code newly documented, overall 24% had an ICD-10 code for esophageal disease, and 13.4% for pulmonary hypertension. The baseline PPV was 78%, which improved to 84% with UTP, identifying 788 patients likely to have SSc. After the ICD-10 code was placed, 63% of patients had a rheumatology office visit. Patients identified by the UTP search algorithm were more likely to have increased healthcare utilization (ICD-10 codes 4 or more times 84.1% vs 61.7%, p < .001), organ involvement (pulmonary hypertension 12.7% vs 6% p = .011) and medication use (mycophenolate use 28.7% vs 11.4%, p < .001) than those identified by the ICD codes alone.

**Conclusion:**

EHRs can be used to identify patients with SSc. Using unstructured text processing keyword searches for SSc clinical manifestations improved the PPV of ICD-10 codes alone and identified a group of patients most likely to have SSc and increased healthcare needs.

## Introduction

Systemic sclerosis (SSc) is a chronic autoimmune and heterogenous disease characterized by microvascular damage, immune dysregulation and multiorgan fibrosis, with skin fibrosis as a distinguishing hallmark [1]. While pathological changes can involve multiple organ systems including the lungs, gastrointestinal tract, kidneys, and heart, the highest disease specific mortality is from associated lung disease, which includes interstitial lung disease (ILD) and pulmonary arterial hypertension (PAH). Together they account for 60% of SSc-related deaths [2], with 10-year mortality for SSc-ILD up to 40% [1]. Timely therapeutic initiation is important, given a signal of skin score benefit with early immunomodulation [3], and stabilization of lung function [4, 5]; the anticipation is that outcomes may be better with earlier intervention [6].

Using electronic health record (EHR)-based cohorts provides several benefits [7] and is valuable for identifying patients with this condition for a variety of reasons. Finding patients in a timely fashion with real-world data can provide better incidence/prevalence estimates of SSc and associated lung disease (ILD, PAH) with less selection bias than in patients enrolled in clinical trials, and this approach may be more likely to include minorities who are underrepresented in prospective cohort studies and trials. EHR-based cohorts also enable examination of vital aspects of clinical care such as screening for ILD [8] and have an extensive history of supporting adverse event monitoring [9]. This offers a unique opportunity for systematic patient monitoring and assessment of risk factor correlation with outcomes.

Only a few studies exist, describing a process of code validation for SSc using EHRs. For example, to identify potential SSc cases, researchers at Vanderbilt University combined clinical data, including laboratory data, and International Classification of Diseases, Ninth/Tenth Revision (ICD-9/10) codes, and reported that the best performing algorithms were a result of using high counts of the code, 4 times or more [10]. Another study performed within the Veterans Health Administration used informatics tools and the Veterans Informatics and Computing Infrastructure (VINCI) to identify SSc patients at risk for scleroderma renal crisis [11]. Laboratory data may not always be performed on every patient, and methods without these data to identify patients with rheumatological disorders perform well in certain instances [12]. Overall, further investigation of algorithms and cohort design with ICD-10 codes and different clinical features is needed for this patient population.

Therefore, we describe a process of building a cohort of patients with newly documented SSc by identifying a combination of ICD-10 codes and keywords in the EHR. We hypothesized that augmenting the ICD-10 codes with selective keywords is a feasible and effective method to reliably detect patients with SSc. We show that the resulting population robustly captures patients with the target condition and is enriched with patients with a higher burden of disease and healthcare utilization. This cohort is intended to enable a series of clinical research investigations leveraging the data-rich EHR.

## Patients and methods

### Study population and variables

**Fig 1:**
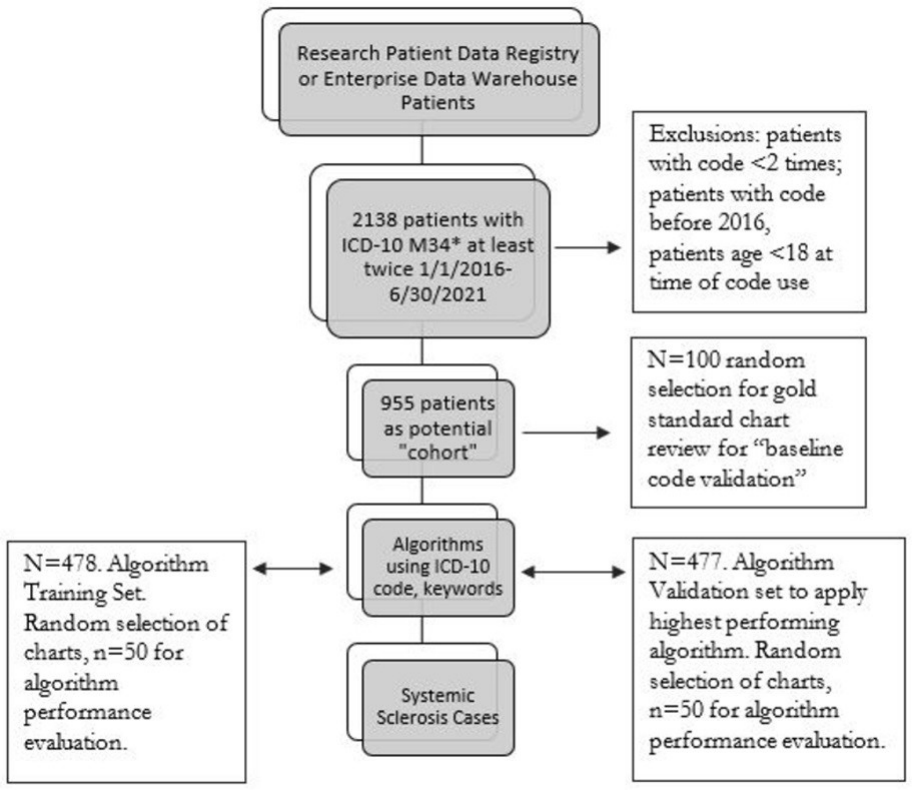
Cohort assembly. Overview of multi-stage process for SSc ICD-10 code validation and algorithm testing and validation. Patients were selected from healthsystem databases if the ICD-10 code was present at least twice in encounters, billing codes or the problem list. Of the 2138 potential patients 1183 were excluded and a random selection of 100 patients underwent code validation. The 955 patient cohort was divided in half for testing of two algorithms using disease manifestation keywords and internal validation of highest performing algorithm. When applied to the entire cohort, 788 patients were identified as likely cases. ICD-10 = International Classification of Diseases, Tenth Revision.

Study participants were sampled from the Mass General Brigham (MGB) patient databases; Research Patient Data Registry (RPDR) and Enterprise Data Warehouse (EDW). Developed in 1980, RPDR is a centralized data registry compiling data from hospitals within the system which now includes longitudinally collected clinical data from over 6.5 million patients [13]. We selected patients with ICD-10 codes of M34* which includes codes for SSc, in encounters, billing and the problem list for the cohort. The most common source was encounters, followed by billing and the problem list. We chose January 1, 2016 as the start date because of routine use of ICD-10 by then, and collected data through June 30, 2021. This identified 2138 unique patients. Based on the general approach that selection of patients with codes applied two times or more results in a more diagnostically accurate cohort [14], we then excluded patients with only one code (n = 485), and also excluded patients who had the code in their chart before 2016 (n = 687) to aim to capture “incident” cases (to reduce likelihood that patients had many years of exposure accrual or time to experience an outcome), resulting in 966 unique patients. The first time the M34* ICD-10 code was documented during the study period was the index date. Of the 966 patients we then excluded 11 patients who were less than18 years old at time of the index date, resulting in 955 adult patients (Fig 1). Structured data elements, included demographics (race/ethnicity extracted from EHR), comorbidities, medications, and laboratory data, were collected along with chart review verification. Organ system involvement was based on selected ICD-10 codes for esophageal disease, Raynaud, pulmonary hypertension [K22.4; I73*; I27.20, I27.29] and medication use was determined by any prescription order available in our system and in the chart during the study period. This study involves human participants but the Mass General Brigham Institutional Review Board exempted this study 2021P001697. Written informed consent and consent for publication not applicable.

### Case definitions and code validation

We developed an abstraction protocol for EHR review, which we used as the gold standard for validating the ICD-10 code and algorithms in the cohort. As a “baseline code validation subset”, a random group of 100 patients were selected from within the cohort for chart review by two pulmonologists (AMT, JR, 50 patients each and blinded to each other’s case determination). To assess reliability, 25 patients of each set were reviewed by the other pulmonologist. Interrater reliability was calculated using the kappa statistic for 50 subjects. Discordant cases were reviewed by a third reviewer (DWB) and discordant non-cases were reviewed by AMT and JR until final consensus was reached. A case was defined as clinician designation stating SSc as a diagnosis, in either the MGB or external system documentation, available for review. Clinician designation was based on a clinical note by a rheumatologist, dermatologist, nephrologist, gastroenterologist, or pulmonologist stating the patient was diagnosed with or likely to have scleroderma, diffuse or limited cutaneous systemic sclerosis (d/lcSSc), or [calcinosis cutis, Raynaud phenomenon, esophageal dysmotility, sclerodactyly, and telangiectasia, (CREST)] syndrome. Additionally, if a physician noted consideration of other diseases (i.e. mixed connective tissue disease [(MCTD]); rheumatoid arthritis ([RA]), Sjogren’s, overlap syndrome) but with SSc features, the subject was considered a case. If there was a report of SSc but no available specialist note, the classification was “missing”. Other classifications included: alternative diagnosis noted in chart (without SSc features or another connective tissue disease noted) and unconfirmed diagnosis (clinician noted diagnosis was uncertain). Charts were also reviewed for data to determine scoring of American College of Rheumatology/European League Against Rheumatism (ACR/EULAR) 2013 classification criteria. Baseline PPV was then calculated from the random selection of 100 charts reviewed and 95% confidence interval presented.

### Algorithm development and validation

Algorithm keyword components were determined based on consensus of a multidisciplinary team of pulmonologists, rheumatologists, and internists. Components were selected based on SSc disease manifestations and accessible data. Among the 955 patients in the cohort, the cohort was divided in half and two unstructured text processing (UTP) searches were run in ambulatory notes, progress notes and discharge summaries for the study period timeframe. RPDR is enabled with note search features using Full Text Search software on the Structured Query Language (SQL) server, which searches documents of different formats from patient databases. Unstructured text processing using tokenization, stemming, stopword removal and the searches are conducted through RPDR and a cohort of patients meeting the search criteria are returned. Among 478 patients in the training set (Fig 1), Algorithm 1: “Raynaud Search” searched for keyword “Raynaud”. Algorithm 2: “Raynaud or Esophageal Search” searched for any one of the keywords “Raynaud, GERD, esophagitis, dysmotility, reflux, esophageal disease”. Using the same case definitions as above, a random selection of 50 charts identified from each algorithm were selected and the chart was reviewed to determine the PPV. For Algorithm 2, a random selection of 50 charts not identified by the algorithm were selected to determine the sensitivity (probability a patient with SSc has the code), specificity, negative predictive value and F-score. Algorithm 2 was then applied to the validation subset of 477 patients and a random subset of 50 charts identified by Algorithm 2 underwent gold standard chart review to determine the PPV.

### Statistical methods

The cohort was partitioned 1:1 for the training and validation subcohorts to allow for an ample sample size available for 10% gold standard chart review, resulting in 50 patients in each subcohort. We evaluated differences between subjects for the total cohort and “baseline code validation” subset using student T-test two-sided for continuous variables, Wilcoxon rank-sum test for nonparametric variables, and Fisher’s exact or chi-square test for binary or categorical variables. Normality of the continuous variables was primarily assessed through visualization of the probability plot in conjunction with application of the central limit theorem for use of T-tests. Normality was additionally evaluated through assessment of skewness, kurtosis, Shapiro-Wilk, and Kolmogorov-Smirnov tests. Similar tests were used for differences between subjects in the case classifications and subjects identified by Algorithm 2. P-values, two-sided, less than 0.05 were considered statistically significant. PPV of each algorithm and 95% confidence interval for each proportion was calculated. Initial data and randomization of training and validation sets used Microsoft Excel (version 2108); analyses were performed using SAS 9.4 (SAS Institute Inc., Cary, NC).

## Results

### Cohort characteristics

The patient cohort included 955 individuals. Table 1 compares the “baseline code validation” subset of 100 patients with the remaining 855 patients. Among the entire cohort, the mean age was 59 years and 84% were female including 84% in the non-validation subset and 78% in the validation subset. The smoking status of the cohort largely consists of unknown (40.5%) or never smokers (33.9%). The cohort was also largely White (74%) with low Charlson Comorbidity Index (3) in both groups. Approximately half of patients in the cohort did not have ANA testing results available in our EHR (54.6%), however among those with testing prior to the ICD-10 code, 96% (218 of 227) were positive and, 89.4% were positive after the ICD-10 code (313 of 350). The extent of SSc organ involvement based on use of ICD-10 code at any time during the study period was 24% for esophageal disease, 22% for Raynaud’s and 13.4% with pulmonary hypertension. There were no statistically significant differences among patients except for slightly higher proportion of patients with pulmonary hypertension in the validation subcohort. During the study period, each year there were between 137–197 patients with the code newly documented in their chart two or more times.

**Table 1:** Characteristics of entire cohort by randomization for baseline code validation subset n = 955.

Characteristic	Non validation subset (n = 855)	Validation subset (n = 100)	P-value
Age at time of diagnosis code, years, mean +/- SD	59.3 +/- 15	59.4 +/- 13.8	.952
Alive, n (%)	776 (90.9%)	86 (86%)	.129
Female, n (%)	721 (84.4%)	78 (78%)	.105
Never Smoker, n (%)	300 (35.1%)	24 (24%)	.163
Former Smoker	189 (22.1%)	24 (24%)	
Current Smoker	27 (3.2%)	4 (4%)	
Unknown	339 (39.7%)	48 (48%)	
White, n (%)	640 (74.9%)	73 (73%)	.145
Black	47 (5.5%)	3 (3%)	
Hispanic	54 (6.3%)	4 (4%)	
Asian	35 (4.1%)	3 (3%)	
American Indian or Alaska Native	2 (0.2%)	0	
Charlson Comorbidity Index Score After Diagnosis	3 (1–4)	3 (2–4)	.604
Median (IQR)			
Malignancy After Diagnosis, n (%)	45 (5.3%)	6 (6%)	.757
ANA Positive During Study, n (%)	432 (91.5%)	44 (89.8%)	.682
Tested N = 472 in nonvalidation; 49 in validation			
Organ Involvement After Diagnosis, n (%)			
• Esophageal disease	156 (18.3%)	21 (21%)	.502
• Raynaud	113 (13.2%)	14 (14%)	.827
• Pulmonary Hypertension	92 (10.8%)	18 (18%)	.032
Treatment During Study Period			
• Prednisone	214 (25.0%)	25 (25%)	.995
• Mycophenolate	219 (25.6%)	26 (26%)	.933
Year of Diagnosis Code			
2016	159 (85%)	28 (15%)	.062
2017	171 (88.6%)	22 (11.4%)	
2018	184 (93.4%)	13 (6.6%)	
2019	165 (90.2%)	18 (9.8%)	
2020	127 (92.7%)	10 (7.3%)	
2021	49 (84.5%)	9 (15.5%)	

SD = standard deviation. ICD-10 = International Classification of Diseases Tenth Revision. IQR = interquartile range. “Unknown” race/ethnicity n = 94, not included.

### Baseline code validation subset characteristics

Of the 50 subjects reviewed by 2 reviewers, 10 discordant cases resulted in a Kappa statistic of 0.46 (only 4 of the 10 were discordant on classification as a case). Based on the random subset of 100 patients for “baseline code validation”, the PPV of the ICD-10 code used two times was 78%. Of the 22 patients with non-case classifications, 9 were classified as unconfirmed, 7 were classified as missing and 6 were classified as alternative. Comparison of 78 SSc cases and 22 patients who were not SSc cases is outlined in Table 2. There was no difference by mean age nor proportion or patients that were female between cases and non-cases, and all non-cases were White or had Unknown race/ethnicity. Of non-cases, 100% were alive, which was significantly more than 82% of cases (p = .036). Among patients classified as cases who had subset documentation, 25% had a notation of CREST/lcSSc in their chart and other common comorbid rheumatological disorders were MCTD (8 patients) and RA (4 patients). Of patients classified as cases, 87% had the ICD-10 code in their chart 4 or more times, as opposed to 59% of the non-cases (p = .006) and were more likely to have Raynaud (18% vs 0%, p = .036). Among non-cases with code use 4 or more times, specific classifications included 6 missing, 5 unconfirmed and 2 alternative. Although not statistically significant, among cases, there were trends toward more esophageal disease (24% vs 9%, p = 0.1) and pulmonary hypertension (21% vs 9%, p = 0.2), and higher likelihood of a rheumatology office visit after the ICD-10 code was placed (63% vs 41%, p = 0.09). Evaluating code and chart review discordance for other common skin conditions, chart documentation of scleredema or other localized skin lesions were 5/300 and 4/300 respectively. Among the 78 patients classified as cases, 58 (74%) had data available with a score greater than 9 for ACR/EULAR criteria. An additional 15 patients had data for consideration of which all had a score of 3 or greater, typically for a combination of Raynaud’s, telangiectasias and/or positive SSc-related autoantibody.

**Table 2:** Characteristics of patients based on case classification N = 100 (baseline validation cohort).

Characteristic	Case	Noncase (alternative, unconfirmed, missing)	P-value
	N = 78	N = 22	
Age, years, mean +/- SD	59.5 +/- 14.4	58.8 +- 12.1	.826
Alive, n (%)	64 (82.1%)	22 (100%)	.036
Female, n (%)	61 (78.2%)	17 (77.2%)	1.000
White, n (%)	56 (71.2%)	17 (77.27%)	.726
Black/African American	3 (3.9%)	0	
Hispanic	4 (5.1%)	0	
Asian	3 (3.9%)	0	
Never Smoker, n (%)	17 (26.9%)	3 (13.6%)	.528
Former Smoker	17 (21.8%)	7 (31.8%)	
Current Smoker	3 (3.85%)	1 (4.6%)	
Unknown	37 (47.4%)	11 (50%)	
ICD-10 code present in problem list per chart review, n (%)	57 (73%)	8 (36.4%)	
ICD-10 code used 4 or more times, n (%)	68 (87%)	13 (59%)	.006
***Clinical Features and Care Utilization***			
ANA Positive Before Diagnosis, n (%) N = 19 tested	16 (20.5%)	3 (13.6%)	.555
Limited SSc or CREST	22 (28.2%)		
Most Common Other Diagnoses			
MCTD	8 (10.3%)		
RA	4 (5.1%)		
Charlson Comorbidity Index Score After Diagnosis	3 (2–5)	2 (2–3)	.284
Median (IQR)			
Malignancy After Diagnosis, n (%)	5 (6.4%)	1 (4.6%)	1.00
Organ Involvement Code After Diagnosis, n (%)			
Esophageal disease	19 (24.3%)	2 (9.1%)	.148
Raynaud	14 (18%)	0	.036
Pulmonary Hypertension	16 (20.5%)	2 (9.1%)	.218
Treatment During Study Period			
Prednisone	21 (26.9%)	4 (18.2%)	.579
Mycophenolate	22 (28.2%)	4 (18.2%)	.419
Rheumatology Office Visit After Diagnosis	49 (62.8%)	9 (40.9%)	.088

SD = standard deviation. ANA = antinuclear antibody. ICD-10 = International Classification of Diseases Tenth Revision. SSc = systemic sclerosis. CREST = calcinosis cutis, Raynaud phenomenon, esophageal dysmotility, sclerodactyly, and telangiectasia. MCTD = mixed connective tissue disease. RA = rheumatoid arthritis. 13 non-cases with code use > = 4 were classified as 6 missing, 5 unconfirmed, 2 alternative.

### Algorithm performance

Of the 478 patients in the training set for algorithm development, search for “Raynaud” (Algorithm 1) resulted in 137 patients (29.1%). Of the 137 patients, code count distribution was 6 with 2 counts, 6 with 3 counts, 125 with > = 4 counts. Among 50 patients randomly selected, 24 had Raynaud only in notes but not on the problem list, thus, using only problem list coding for Raynaud could miss cases; of these 24 patients only one was not classified as a case. Overall, among those with a SSc subset described, 25 of 50 (50%) patients were noted to have lcSSc/CREST. The PPV for Algorithm 1 was 84%. Algorithm 2: “Raynaud+Esophageal Search” resulted in 375 patients (78%). Of the 375 patients, code count distribution was 22 with 2 counts, 33 with 3 counts, and 320 with > = 4 counts. Among 50 patients randomly selected with a SSc subset described, 22 patients were noted to have lcSSc/CREST (44%). In all, 26 patients had the keywords in notes but not their problem list. Within the training set, Algorithm 2 was further analyzed to determine multiple performance characteristics, outlined in Table 3. This resulted in 58% sensitivity, 70% specificity, 38% NPV, 84% PPV and an F-score of 68. We then applied Algorithm 2 to the validation subset of 477 patients, identifying 394 patients (82.6%), with a PPV of 76%. Using Algorithm 2 on the entire cohort, we identified 788 patients likely to have SSc. Of the 167 patients not identified, 14 were classified as cases from the “baseline code validation” subset. Within the entire cohort, to determine if Algorithm 2 distinguishes a subpopulation of patients different from those with the ICD-10 code alone but not identified from the algorithm, select comparisons are highlighted in Table 4. There are statistically significantly differences between patients for use of the code > = 4 times (84.1% vs 61.7%, p < .001), pulmonary hypertension (12.7% vs 6%, p < .011), and a rheumatology office visit after the code use (66.9% vs 44.9%, p < .001).

**Table 3:** Performance characteristics of ICD-10 code and algorithms.

ICD-10 code or Algorithm	PPV (95% CI)	Sensitivity^d^	Specificity^d^	Negative Predictive Value	F-score^d^
ICD-10 code > = 2^a^	78 (.70, .86)				
Algorithm 1 Training Subset	84 (.74, .94) ^**c**^				
N = 137 identified ^**b**^					
Algorithm 2 Training Subset	84 (.74, .94) ^**c**^	58	70	38	68
N = 375 identified ^**b**^					
Algorithm 2 Validation Subset	76 (.64, .88) ^**c**^				
N = 394 identified					

ICD-10 = International Classification of Diseases Tenth Revision. PPV = positive predictive value.

^a^Baseline case validation n = 100 random selection

^b^N = out of 478 training subset

^c^N = 50 random selection for subjects identified by algorithm.

^d^N = 100, full characteristics calculated using additional random selection of 50 patients not identified by algorithm

**Table 4:** Select comparisons of patients identified by Algorithm 2 compared to patients with ICD-10 code use at least two times.

Characteristic	Identified by Algorithm 2 n = 788	Not Identified by Algorithm 2 n = 167	P-value
ICD-10 code used 4 or more times, n (%)	663 (84.1%)	103 (61.7%)	< .001
Prednisone Use During Study, n (%)	207 (26.2%)	32 (19.2%)	.061
Mycophenolate Use During Study, n (%)	226 (28.7%)	19 (11.4%)	< .001
Organ Involvement Code After Diagnosis, n (%)	100 (12.7%)	10 (6%)	.011
Pulmonary Hypertension			
Rheumatology Office Visit After Diagnosis n (%)	527 (66.9%)	75 (44.9%)	< .001

ICD-10 = International Classification of Diseases Tenth Revision.

## Discussion

Systemic sclerosis is a very uncommon diagnosis with substantial morbidity and mortality, and diagnostic evaluation can be difficult. Electronic health records are now widely used in the U.S. and other developed countries and represent a valuable tool for identifying conditions such as this. Creating algorithms for easier and reliable identification of patients is important in aiding clinical research for this patient population with high morbidity and mortality, especially from lung disease [15]. We utilized data from EHRs to assemble a cohort of patients with systemic sclerosis. Specifically, we analyzed the reliability of the ICD-10 code documented twice to correctly identify SSc cases, and then created algorithms to improve the test characteristics and evaluate the frequency of disease coding over a five-year interval. Our approach included ICD-10 codes, clinical data and incorporation of UTP retrieval terms to enhance identification, without the need for laboratory data as a feature. In our study, the baseline PPV for the M34* ICD-10 code applied two or more times was high and improved further with UTP using keywords related to organ manifestations. These PPV over 70 are generally accepted as high [16].

Prior studies validating ICD codes for SSc examined ICD-9 [17], ICD-10 [18] or both, and use a variety of case definitions: ACR/EULAR classification criteria and/or clinician documentation [19]. We elected to use expert physician designation to determine classification of cases because this is a commonly used practice [10, 11] especially as all the information to fully assess criteria may not be available. In one study, the PPV for the ICD-10 code ≥2 times was 84% [10], and for a series of ICD-10 codes used as a discharge diagnosis was 88–100% [18]. Assessment of the code when used as a primary discharge diagnosis is likely to skew towards higher PPV because this would reflect the primary reason for a hospitalization, which is more likely to be reliably coded. Jamian et al suggested that gastrointestinal keywords would be nonspecific and might result in an algorithm capturing patients without SSc [10], however we demonstrate adding them to ICD-10 codes resulted in identification of more patients and the same PPV as using Raynaud only. This resulted in an F-score (68) which was similar to their study using ICD-10 and ANA [10]. Focusing on the high specificity and PPV of Algorithm 2 reassures that the patient population identified will likely have the disease. Our algorithms offer SSc clinicians and researchers the ability to construct cohorts for clinical monitoring (for example, medication impact on organ specific activity) or clinical research (for example, lung disease incidence or patient reported outcomes), while still preserving other data elements as possible predictors. Applying Algorithm 2 to our cohort resulted in 788 possible SSc cases, which are distinguished from patients with baseline entry requirement of the code 2 times or more and not identified by the algorithm, by multiple code use, followup, and development of pulmonary hypertension. This characterizes the population captured by the algorithm as distinct in care utilization and development of further organ involvement.

Analyses obtained in the study have implications for cohort design and assessment. Gold standard chart review revealed about half of patients may not have SSc on their problem list, therefore, relying solely on codes from the problem list could limit retrieval of patients in other health systems. Reasons for discordance of the ICD-10 code and chart review classification as a non-case were varied. Some subjects were primarily managed external to our healthcare system therefore they did not have readily available notes. For some subjects, the code was placed during initial suspicion for SSc which was ultimately deemed less likely, but the code may not have been removed. It is interesting that 59% of non-cases in the baseline code validation set had codes used 4 times or more in their chart, which may be a function of visits for subspecialty evaluation or during the initial diagnostic workup.

The ultimate SSc cohort that was established by at least two documentation instances of ICD-10 codes is like others in some ways but differs in others. Over the study period, approximately 175 patients a year have the ICD-10 code newly placed in their chart, this frequency may be a function of the role as a referral hospital. Algorithms may perform differently if the underlying disease prevalence in a healthsystem is different [16]. In this cohort, 75% of patients are White, and while some data suggests there may be increased prevalence among Black patients [20] this was not captured in our healthcare system, possibly be due to care delivery patterns. Overall, this cohort of patients is sociodemographically similar to other studies of patients with SSc [10]. The described clinical characteristics of the 955 patients may differ from expected clinical patterns for several reasons. Selecting specifically for mycophenolate and prednisone, our utilization is like other studies [21] initially identifying patients by ICD-10 code. Non-cases may have medication orders because they have other connective tissue disease indications for use. Although most patients with SSc have Raynaud’s, the overall low rate of coding for Raynaud in our cohort (22% overall (which includes non SSc cases); Algorithm 1 also only resulted in 29% of patients of the testing subset) may not be unexpected for this specific disease feature, especially if a code for SSc is already used, or a visit/encounter was for another specific organ-based indication. This is similar in other disease states, such as ocular disease [22], when more general, rather than organ specific codes are used; this could also be expected for esophageal disease coding as well. Among those with a documented SSc subset, chart review during baseline cohort validation and Algorithms 1 and 2 found limited SSc/CREST documented for 28%, 50% and 44% of patients respectively.

The main strength of this study that we have designed an automated approach for accurately identifying patients with SSc through the EHR, creating a cohort which can be used in a variety of ways. The exclusion of laboratory and medication data in case definitions overcomes problems with these data which may not be performed or require data transformation, and this approach also allows for inclusion of patients with possibly mild disease not yet on therapy. However, this approach also has limitations. The algorithm is dependent on available data; if patients have predominantly external care and are referred for targeted evaluation, there may not be enough text data for review. Sampling errors may also exist with a random selection of charts for gold standard chart review. Using subspecialist note as criteria for case definition could also compound inequities if patients from minoritized backgrounds are less likely to have subspecialty referral/care but could have other clinician notes regarding the diagnosis. We aimed to capture “incident” cases by excluding subjects who had the code prior to our study entry date January 2016, however, some subjects were documented to have SSc diagnosis earlier than the first date the ICD-10 code was applied, which is not an uncommon challenge with proxy dates, that there may be delay between disease onset, recognition and ultimate coding. There are many ways to define a disease diagnosis date in the EHR but how it reflects disease onset date is a universal challenge. External validation in another health system using this approach would further evaluate the utility and performance of these algorithms.

There is a powerful role for data gathered from the EHR to develop hypotheses, link data sources, expand analyses and advance SSc care [23]. To our knowledge, this is the first study to evaluate accuracy of collective SSc M34*ICD-10 codes extracted from a variety of clinical scenarios (billing codes, encounters and problem list) within the EHR and the use of UTP algorithms to refine the cohort based on keywords of organ system involvement including esophageal disease or gastrointestinal symptoms. Future studies can include developing an algorithm for patients without SSc codes to determine likelihood of missed cases in our system which could improve diagnostic efficacy. In conclusion, we created high performing algorithms using a combination of ICD-10 codes and clinical keywords to accurately identify patients in our health system with SSc. This method has considerable human resource advantages compared to manual chart review and identifies a high-utilization patient population compared to use of the ICD-10 code alone. This work demonstrates how the EHR can be leveraged to create cohorts designed to allow clinical research investigation.
